# Montmorillonite-Sodium Alginate Oral Colon-Targeting Microcapsule Design for WGX-50 Encapsulation and Controlled Release in Gastro-Intestinal Tract

**DOI:** 10.3390/jfb15010003

**Published:** 2023-12-19

**Authors:** Yibei Jiang, Zhou Wang, Ke Cao, Lu Xia, Dongqing Wei, Yi Zhang

**Affiliations:** 1 Department of Inorganic Materials, School of Minerals Processing and Bioengineering, Central South University, Changsha 410083, China; 215611016@csu.edu.cn (Y.J.); zhouwang@csu.edu.cn (Z.W.); 2 Department of Oncology, The Third Xiangya Hospital of Central South University, Changsha 410078, China; csucaoke@163.com; 3 Center for Medical Genetics & Hunan Key Laboratory of Medical Genetics, School of Life Sciences, Central South University, Changsha 410078, China; xialu@sklmg.edu.cn; 4 State Key Laboratory of Microbial Metabolism, School of Life Sciences and Biotechnology, Shanghai Jiao Tong University, Shanghai 200240, China

**Keywords:** montmorillonite, sodium alginate, microcapsule, controlled drug release, oral administration

## Abstract

The montmorillonite-sodium alginate (MMT-SA) colon-targeting microcapsules have been designed as a WGX-50 encapsulation and controlled release vehicle used in oral administration. The MMT-SA microcapsule was formed from a cross-linking reaction, and the stable micropore in the microcapsule changed with a different MMT-SA mixed mass ratio. The MMT-SA microcapsule has a reinforced micropore structure and an enhanced swell–dissolution in SIF and SCF with alkaline environment, which is attributed to the incorporated MMT. The MMT-SA microcapsule exhibited a high WGX-50 encapsulation rate up to 98.81 ± 0.31% and an obvious WGX-50 controlled release in the simulated digestive fluid in vitro. The WGX-50 loaded with MMT-SA microcapsule showed a weak minimizing drug loss in SGF (Simulated Gastric Fluid) with an acidic environment, while it showed a strong maximizing drug release in SIF (Simulated Intestinal Fluid) and SCF (Simulated Colonic Fluid) with an alkaline environment. These features make the MMT-SA microcapsule a nominated vehicle for colon disease treatment used in oral administration.

## 1. Introduction

Drugs for oral administration with controlled release used in the digestive tract result in long-term stable release in large doses and have a high mass fraction therapeutic effect in target-specific sites [1]. There are some unique characteristics that should be taken into account, such as drug transport across the biologic barriers, drug release in the digestive tract microenvironment, and the drug molecule’s absorption, distribution, metabolism, and excretion in the digestive tract, etc. [2,3,4]. WGX-50 is an amide compound extracted from Zanthoxylum Bungeanum Maxim, exhibiting a beneficial therapeutic effect on colon disease management through modulating the innate immune response. WGX-50 exhibits swift oral absorption, posing challenges in achieving uniform systemic distribution and targeted delivery to the colonic site, thereby limiting its therapeutic efficacy. Furthermore, the acidic milieu of the stomach may induce structural modifications in WGX-50. WGX-50 in oral administration should avoid an uncontrollable release in an inconvenient location [5,6,7,8]. Therefore, a WGX-50 encapsulation and controlled release vehicle used in oral administration should be designed for minimizing drug loss within the gastric tract microenvironment and accelerating drug absorption in the intestinal tract, and further facilitating therapeutic effects in the target-specific site [9,10,11,12,13].

Sodium alginate (SA) hydrogel microcapsules formed from an SA solution in various cross-linking reactions are water-swollen biomaterial with a three-dimensional network structure that exhibit remarkable features, such as a tunable microporous structure, non-toxicity, biodegradable and biocompatible, etc., which could be used as a drug encapsulation and controlled release vehicle used in oral administration [14,15,16,17]. Several studies on functionalized hydrogel microcapsules with enhanced mechanical structures for sustained and controlled drug release have been conducted, but undesired leakage makes them inefficient, i.e., encapsulating the drug molecule in a reinforced micropore structured microcapsule [18,19,20]. Montmorillonite (MMT) is a US Food and Drug Administration-approved active and inactive ingredient for diverse biomedical applications owing to its characteristics such as its two-dimensional structure in micronano scale and dual-charged distribution in structure while having a net negative charge in aqueous suspension, its high swelling behavior and inherent stiffness, non-toxicity and biocompatibility, etc. [21]. Hence, our foremost consideration revolves around the controlled release of the pharmaceutical agent and ensuring the safety and stability of the carrier. The hydrogel microcapsule functionalized with MMT-SA as a reinforced vehicle against chemical issues from the digestive tract microenvironment and even accelerating the drug molecule’s absorption, distribution, metabolism, and excretion in the intestinal tract is an essential choice for drug encapsulation and controlled release vehicle used in oral administration [22,23].

The novelty of this work is that an oral colon-specific drug delivery system (MMT-SA microcapsules) has been designed taking into account the digestive tract microenvironment, which could benefit from targeted controlled release, efficient delivery, and even a reduction in initial losses for WGX-50. The microcapsule formation mechanism and the main influence factors at different stages are discussed in detail. The ratio of MMT to SA was adjusted to obtain microcapsules with optimal particle size, pore distribution, and thermal stability. The cytotoxicity of the microcapsules towards human cell lines associated with colon diseases has been evaluated. Additionally, the storage and release of WGX-50 in simulated digestive fluid in vitro provide an enhanced verification model in certain colon disease treatments.

## 2. Materials and Methods

### 2.1. Materials

Medical Montmorillonite (MMT, 98%, Product No.: SD1004) was obtained from Sand Technology Co., Ltd. (Ezhou City, China) Sodium alginate (SA, AR, 90%, CAS No.: 9005-38-3) was obtained from Maclin Biochemical Technology Co., Ltd. (Shanghai, China). Calcium chloride (AR, 96%, CAS No.: 1004-52-4) was purchased from Sinopharm Chemical Reagent Co., Ltd. (Shanghai, China). WGX-50 (MW: 311.384, CAS No.: 29946-61-0) was obtained from Sigma-Aldrich (Shanghai, China).

### 2.2. Preparation of MMT-SA Microcapsule

From the working solutions, MMT and SA were mixed with different mass ratios in 10 mL aqueous solution and stirred at room temperature. The mixing was carried out with a mixing time of 60 min and a mixing rate of 500 rpm, to ensure thorough mixing. Later, the mixed suspension was added to a 0.4 M CaCl_2_ solution, and the 30 min cross-linking was there to ensure the completed reaction, i.e., the cross-linking reaction time is 30 min. Then, the microspheres were collected through filtration and washed with deionized water three times. Liquid nitrogen freeze-drying was used to ensure microsphere transformed into a microcapsule. The mixed mass ratios for the MMT-SA microcapsule to obtain different phases are presented in Supplementary Material Table S1.

### 2.3. Characterization

SEM images were evaluated using a Mira3 LMU SEM (Tescan, Brno, The Czech Republic) with a vacuum set to below 5 × 10^−3^ Pa, accelerating voltage was 20 kV, and spot size was 5.5–6.5 nm. Platinum was used as the gold-spraying material and the gold-spraying time was set to 120 s to increase the conductivity of the samples. The differential thermal analysis was analyzed using the STA449C (NETZSCH Machinery and Instruments, Selb, Germany), which ranged from 30 to 600 °C under constant argon purging with 10 °C/min rate of rise. The zeta potential values were measured using a Malvern Zetasizer Nano S90 (Malvern Instruments, Malvern, UK). The gel precursor, dissolved in buffer solutions with varying pH levels, was placed in a quartz cuvette. The values of zeta potential were obtained from three measurements. The diffuse reflectance spectrum was measured using a Fourier transform infrared spectrometer (Shimadzu FTIR 8120 spectrometer, SHIMADZU, Kyoto, Japan) in the range from 400 to 4000 cm^−1^.

### 2.4. Cell Culture and Cytotoxicity Evaluation

Human intestinal epithelial cells 6 (HIEC-6) and human colonic epithelial cells (NCM460) were obtained from Abiowell Biotechnology Co., Ltd. (Changsha, China). HIEC-6 and NCM460 cells were cultured in RPMI 1640 medium containing 10% FBS and 1% Pen/Strep at 37 °C with 5% CO_2_ atmosphere.

The culture medium was removed and replenished with RPMI 1640 culturing medium containing MMT-SA mixture ranging from 1 μg/mL to 300 μg/mL, and then incubated for 24 h at 37 °C. The viable cell counts were measured using Cell Counting Kit-8 (CCK-8) according to the manufacturer’s instructions (Beijing Lablead Biotech, CK001-3000T, Beijing, China). The absorbance at 450 nm was measured using BIOTEK ELX800 Universal Microplate Reader (Thermo Fisher Scientific, Waltham, MA, USA).

### 2.5. Drug Encapsulation Measurement

WGX-50 with amounts ranging from 25 mg to 200 mg was dissolved in the aforementioned 10 mL MMT-SA initial mixed solution, and then stirred at room temperature for 60 min. The resulting mixture was added into 40 mL 0.4 M CaCl_2_ solution in 30 min. The MMT-SA microcapsule containing WGX-50 (WGX-50/MMT-SA) was filtered and washed three times with deionized water to remove the residual WGX-50 on the microcapsule surface. The absorbance of the residual WGX-50 at 277.5 nm was measured using a UV-Vis spectrophotometer (UV2600, Thermo Fisher Scientific, Waltham, MA, USA).

The encapsulation rate (EE, %) and the drug loading rate (DL, %) were calculated using the following Equations (1) and (2) [24]:

(1) $$EE(\%)=(W_{Fed}-W_{Non-encapsulated})/W_{Fed}\times 100\%$$

(2) $$DL(\%)={(W}_{Fed}-W_{Non-encapsulated})/W_{Total}\times 100\%$$

where W_Fed_ is the initial WGX-50 amount in total, W_Non-encapsulated_ is residual WGX-50 amount in filtrate, and W_Total_ is the microcapsule amount containing WGX-50.

### 2.6. Drug Release In Vitro

In vitro WGX-50 release experiments were carried out in an alkaline environment (pH 6.8 in PBS solution, Simulated Intestinal Fluid, SIF; pH 7.4 in PBS solution, Simulated Colonic Fluid, SCF) and in an acidic environment (pH 1.2 in PBS solution, Simulated Gastric Fluid, SGF). To simulate the sequential pH changes that occur during the in vivo process, an in vitro release study mimicking the gastrointestinal tract was conducted for 30 h. Here, 30 mg WGX-50/MMT-SA microcapsules were sequentially immersed in 200 mL of SCF, SIF, and SGF for 2 h, 3 h, and 24 h at 37 °C 100 rpm. Then, 5 mL suspension was centrifuged at 3000 rpm in selected time intervals, the supernatants were measured using UV-Vis (λ = 277.5 nm) spectrophotometer, and the sediments were redispersed in 5 mL PBS solution to replenish the total volume. All the experiments were performed at least in triplicate.

## 3. Results and Discussion

### 3.1. Fabrication of the MMT-SA Microcapsule

**Figure 1:**
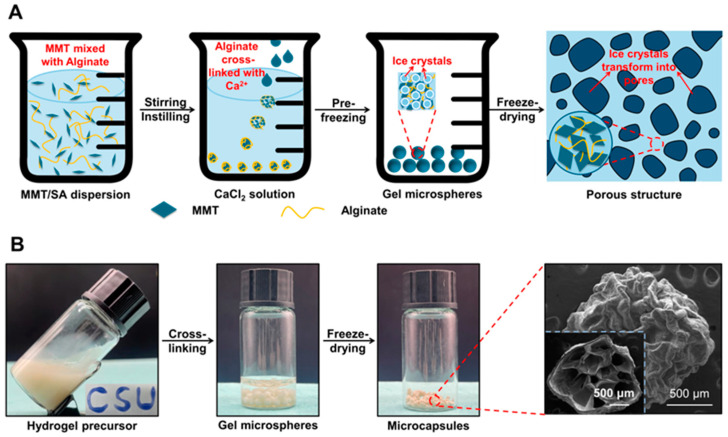
(**A**) Schematic illustration, (**B**) digital photographs and the representative SEM images for MMT-SA microcapsules.

Schematic illustration, digital photographs, and the representative SEM images for MMT-SA microcapsules are shown in Figure 1. The MMT was mixed with the SA solution, and the resulting mixed solution is a turbid colloidal suspension with viscosity and opacity, indicating that the MMTs were well scattered within the SA solution without obvious aggregation. Moreover, the mixed turbid colloidal suspension was flexible and stretchable, allowing it to be shaped into letters. This property is beneficial for fabricating hydrogel microspheres with a stable microstructure. Later, the mixed solution was added with the calcium chloride solution to form hydrogel microspheres, and the initial hydrogel microsphere exhibited opacity and hydrophobicity. Subsequently, the microcapsule was formed from freeze-dried hydrogel microspheres, and the irregular micropores in the microcapsule were created through the sublimation of ice crystals. Those microcapsules exhibited near-spherical shape in appearance and distinct ripple-like cell walls with a clear skeleton structure in their internal structure. Additionally, the oven-dried microcapsules have a smoother surface but lacked micropores compared to the freeze-dried microcapsules (Figure S1).

### 3.2. Different Phases of the MMT-SA Microcapsule

**Figure 2:**
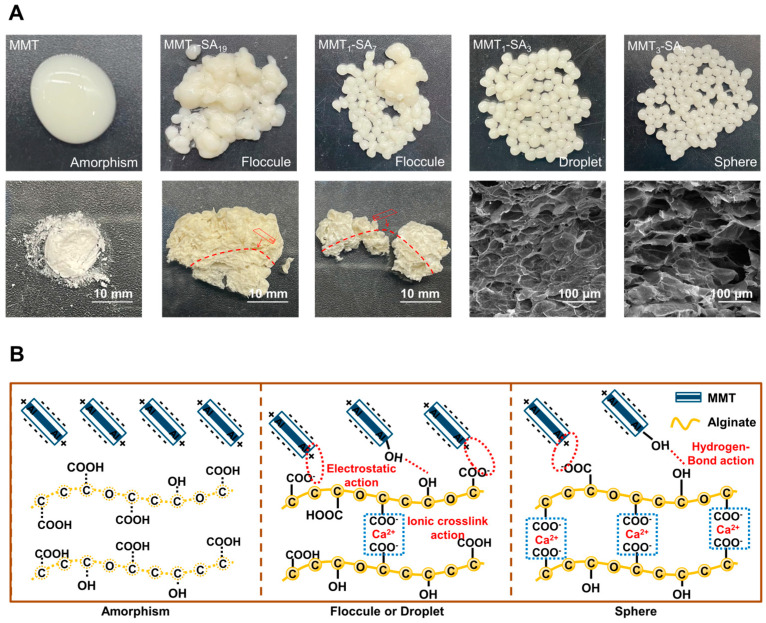
(**A**) The digital photographs, SEM images, and (**B**) the corresponding schematic illustration for MMT-SA microcapsule with different mixed mass ratios.

The schematic illustration and the corresponding digital photographs and SEM images for MMT-SA microcapsule with different phases are shown in Figure 2. Figure 2A illustrates MMT and SA with different mixed mass ratios, resulting in various states, ranging from a mixed turbid colloidal suspension to a stable spherical microcapsule. The stable spherical microcapsules exhibited a smooth shell in wet state and uniform micropores in freeze-dried microspheres, which is beneficial to enhance drug encapsulation and controlled release in target-specific sites [25]. Additionally, the MMT agglomerates in the stable walls are presented in Figure S1. FTIR was used to record the characteristic peaks in the MMT-SA microcapsule in Figure S2. Peaks at 3485 cm^−1^, 2925 cm^−1^, 1612 cm^−1^, 1417 cm^−1^, and 821 cm^−1^ were attributed to SA [26]. Peaks at 3629 cm^−1^, 3417 cm^−1^, 1639 cm^−1^, 1432 cm^−1^, and 1030 cm^−1^ were attributed to MMT [27]. Peak at 1030 cm^−1^ shifting to 1040 cm^−1^ indicated a cross-linking reaction between MMT and SA. The TG–DSC curves of MMT (Figure S3A) exhibited endothermic peaks at 98 °C and 369 °C, indicating the loss of physically absorbed water molecules and the elimination of the water lattice in the compound. The thermal degradation of the MMT-SA composite ranged from 13.82% to 16.45% at temperatures between 121 °C and 137 °C, with an observed endothermic peak at 136 °C attributed to the removal of adsorbed surface water moisture in the composite (Figure S3B–E). The exothermic peak observed at temperatures ranging from 231 °C to 287 °C corresponds to the breakdown of alginate chains in the hybrid composite, as evidenced by its absence in the DSC curve of unblended MMT [28,29]. Another thermal decomposition was observed between 752 °C and 757 °C due to volatilization of residual SA carbons (Figure S3C–E) [30].

The MMT-SA microcapsule was fabricated using a cross-linking technique, as shown in Figure 2B. The layered MMT consists of one Al-octahedral sheet sandwiched between two opposing Si-tetrahedral sheets. The hydroxyl groups in silanol (Si–OH) and/or aluminol (Al–OH) groups existed in the internal, the external, and the edge surface areas. MMT’s total negative charge originates from constant charge from the isomorphic substitution-induced charge compensating cations, variable charge from the active groups on the surface, and an opposite charge from various exchangeable cations [31]. SA consists of (1, 4)-linked β-D-mannuronic acid and α-L-guluronic acid units. There are abundant carboxyl groups along its backbone, which could be used to connect to other compounds. SA’s total negative charge originates from protonated carboxyl groups in acidic conditions [32]. SA interacted with MMT to form complexes through anion–cation interaction and electrostatic attraction and absorption, which was almost simultaneous with the cross-linking reaction. It should be noted that multiple factors in SA absorbed on the MMT surface were not discussed in this manuscript, such as functional groups in SA, exchangeable cations in MMT, and charge value in all components, etc. Moreover, SA did not undergo intercalation into MMT.

Insufficient SA amount resulted in an inadequate cross-linking reaction, leading to the formation of an unstably structured microsphere that disintegrated with a slight disturbance or deformation with a changing environment. With an increased SA amount, the abundant COO^−^ from SA reacts with the Ca^2+^ from the CaCl_2_ solution to form a cross-linked Ca-alginate. The cross-linking reaction is not controlled due to the CaCl_2_ solution being water-soluble. The stable structured microsphere with a three-dimensional network structure has an obvious advantage in minimizing drug leakage within the digestive tract microenvironment [33].

### 3.3. Different Mixed Mass Ratios for the MMT-SA Microcapsule

**Figure 3:**
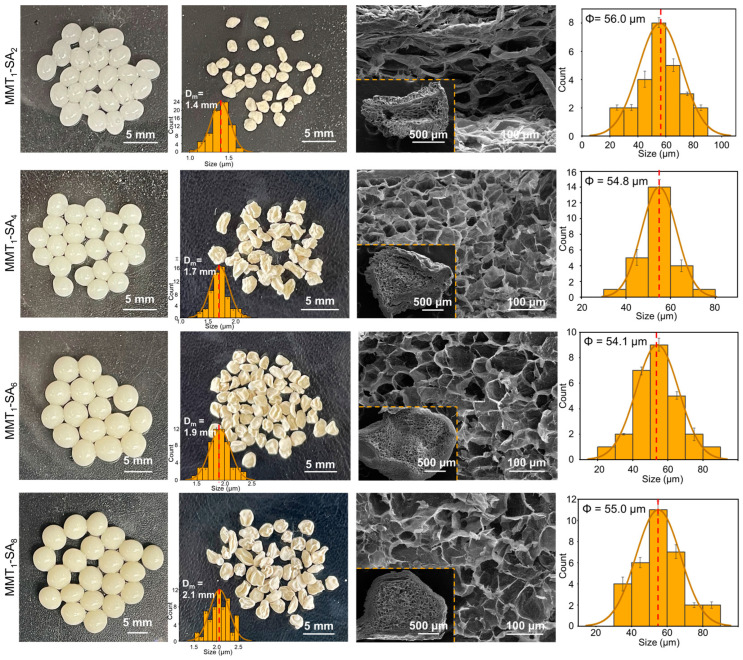
Digital photographs, the cross-sectional SEM images, and the corresponding size distribution for MMT-SA microcapsule with different mixed mass ratios.

Digital photographs, the cross-sectional SEM images, and the corresponding size distribution of the MMT-SA microcapsules with different mixed mass ratios are shown in Figure 3. The stable spherical microcapsule exhibited a smooth shell and was non-transparent white in a wet state and shrunken and wrinkled in appearance in a freeze-dried state, while exhibiting uniform micropores as a freeze-dried microsphere. As the MMT amount increases, the stable spherical microcapsule exhibits a darker appearance in the wet state, a larger size in the freeze-dried state from 1.4 mm to 2.1 mm, and a more intact and serried microcellular structure of the freeze-dried microsphere. The MMT-SA microcapsule with mass ratio of 300:75 was selected as the optimized drug-encapsulation and controlled-release vehicle used in oral administration, which resulted from overall considerations og MMT’s toxic effects mentioned in the literature and the good microcellular structure in this condition [34].

Additionally, the MMT-SA microcapsule showed a negative charge as shown in Figure S4 and indicated that it could be adsorbed on the inflamed tissue in the intestinal tract through electrostatic interaction [35]. Due to certain biological differences between humans and other animals, the use of human-derived cells can better mimic biological processes in the human body and, thus, more accurately assess the safety and efficacy of drugs. The MMT-SA microcapsule was non-toxic towards the intestinal drug absorption-associated cells (human intestinal epithelial cells, HIEC-6 cells and human colonic epithelial cells, NCM460 cells) as shown in Figure S5, according to the ISO-10993 Standard [36,37].

### 3.4. Drug Encapsulation and Release In Vitro for the WGX-50 Coated with MMT-SA Microcapsule

**Figure 4:**
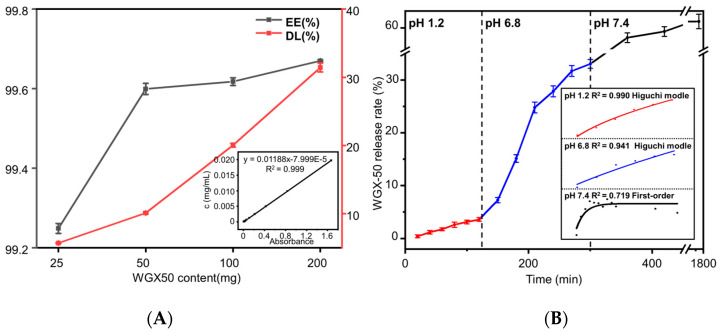
The WGX-50 encapsulation, where x is the absorbance (**A**) and release (**B**) in vitro for the WGX-50/MMT-SA microcapsule.

The WGX-50 encapsulation and release in vitro for the WGX-50/MMT-SA microcapsule is shown in Figure 4. The WGX-50 encapsulation rate and loading rate is shown in Figure 4A, and the WGX-50 concentration calibration curve is shown in the lower-right corner. The WGX-50 encapsulation rate reached more than 90% when the WGX-50 amount was under 50 mg and followed a slow growth in WGX-50 encapsulation rate with an increasing WGX-50 amount. Hence, the optimal initial WGX-50 amount was 50 mg according to the cost-effectiveness research, and the WGX-50 encapsulation rate in the MMT-SA microcapsule was 99.60%.

FTIR was used to record the characteristic peaks of the WGX-50/MMT-SA microcapsule in Figure S6. Peaks at 1657 cm^−1^, 1534 cm^−1^, 1513 cm^−1^, 1330 cm^−1^, 1280 cm^−1^, and 1260 cm^−1^ were attributed to WGX-50. Peaks at 1657 cm^−1^, 1534 cm^−1^, and 1513 cm^−1^ in the WGX-50/MMT-SA microcapsule indicated that WGX-50 was encapsulated in MMT-SA microcapsule with surface adsorption rather than chemical bonding, which was beneficial for drug encapsulation and controlled release. To investigate the phase transition characteristics of the WGX-50/MMT-SA microcapsule, TG-DSC analyses were conducted, as shown in Figure S7. The mass loss of WGX-50 occurred within the temperature range of 280–440 °C, indicating the complete degradation of the compound. The endothermic peaks observed at 130 °C and 404 °C correspond to the melting point and decomposition of the crystalline WGX-50, respectively. However, in the DSC traces of WGX-50/MMT-SA, the sharp peak associated with crystalline WGX-50 was no longer present. This suggests that the WGX-50 lost its crystal structure during the encapsulation process within the MMT-SA composite, transforming into an amorphous state within the microcapsules [38].

WGX-50 release behavior from the WGX-50/MMT-SA microcapsule in simulated digestive fluid in vitro is shown in Figure 4B. The WGX-50/MMT-SA microcapsule showed a controlled fast release (32.42% and 61.66%) in alkaline environment (Simulated Intestinal Fluid, SIF; Simulated Colonic Fluid, SCF), followed by a snail-like slow release (3.46%) in an acidic environment (Simulated Gastric Fluid, SGF). WGX-50 release behavior from the WGX-50/MMT-SA microcapsule in SGF and SIF seems to fit the Higuchi model, and the release mechanism is Fick diffusion, indicating that WGX-50 adsorbed on the shell surface in small amounts while stored within the microcapsule in large amounts. The WGX-50 adsorbed on the shell surface could affect monitoring the adverse reactions but do not affect the therapeutic effects. The WGX-50 release behavior from WGX-50/MMT-SA microcapsule in SCF seems to fit with the First-order model, and the WGX-50 release rate correlates with initial WGX-50 concentration [39]. Additionally, the release component n, the rate constant k, and the correlation coefficient R^2^ for the WGX-50/MMT-SA microcapsule in simulated digestive fluid in vitro are listed in Table S3.

### 3.5. Drug Release Mechanism and the Swell–Dissolution Model for the WGX50 Coated with MMT-SA Microcapsule

**Figure 5:**
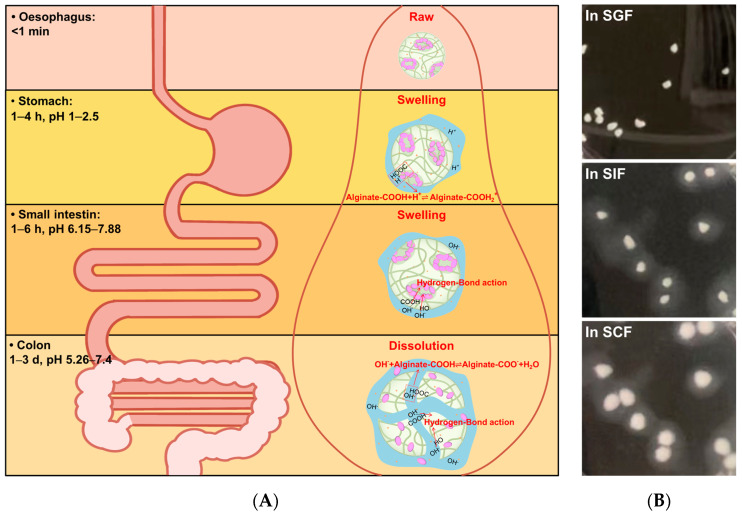
The WGX-50 release mechanism (**A**) and the swell–dissolution model (**B**) for WGX-50/MMT-SA microcapsule.

The WGX-50 release mechanism and the swell–dissolution model for the WGX-50/MMT-SA microcapsule are discussed in Figure 5. In SGF with an acidic environment, the microcapsule structure was compact with a low swelling rate. This is attributed to the –COOH of alginate being partially protonated and forming hydrogen bonds. The formation of hydrogen bonds leads to increased intermolecular interactions, resulting in a denser molecular network, making it more difficult for water molecules to enter the interior of the microcapsule, thus reducing the swelling rate of the microcapsule.

The total WGX50 slow release consists of surface desorption from WGX-50 adsorbed on the microcapsule surface and microporous diffusion from WGX-50 stored within the microcapsule. In SIF and SCF with an alkaline environment, the microcapsule structure was loose with a high swelling rate. This phenomenon can be attributed to the increased levels of OH^−^ interacting with the –COOH and –OH within the microcapsules to form hydrogen bonds, thereby weakening the intermolecular interactions and reducing the degree of coiling and entanglement of the calcium alginate molecular chains, resulting in a looser microcapsule structure and increased swelling [40]. Additionally, MMT could accelerate the water molecules’ absorption, resulting in further swelling and dissolution of the MMT-SA microcapsule. The total WGX-50 burst release was attributed to the alkali corrosion and microcapsule dissolution. Hence, the structural construction of the microcapsule is beneficial for drug encapsulation and controlled release at a target-specific site.

## 4. Conclusions

In this study, we successfully prepared MMT-SA microcapsules with different ratios as a delivery system for WGX-50, aiming to enhance the drug’s bioavailability. The addition of MMT as a filler enabled the formation of spherical microcapsules with a dense and uniform internal pore structure. The successful encapsulation of WGX-50 within the microcapsules was confirmed through FTIR analysis. The WGX-50 loaded with MMT-SA microcapsules exhibited minimal drug loss in the acidic environment of SGF, while demonstrating significant drug release in the alkaline environments of SIF and SCF. MMT-SA microcapsules are considered to be an ideal drug-encapsulation and controlled-release vehicle for oral treatment of colon diseases due to their good biosafety for human gastrointestinal cells (HIEC-6 cells and NCM460 cells) as well as the improved microporous structure with enhanced swell–dissolution properties in SIF and SCF (alkaline environment). And then, the in-depth discussion on the absorption, distribution, metabolism, and excretion of the drug molecule in the digestive tract, etc., is a starting point for the following work.

## Data Availability

The data presented in this study are available on request from the corresponding author.

## Supplementary Materials

The following supporting information can be downloaded at: https://www.mdpi.com/article/10.3390/jfb15010003/s1. Figure S1. Freeze-dried microcapsules (A-B) and oven-dried microcapsules (C-D). Figure S2. FTIR of SA, MMT, MMT_1_-SA_3_, MMT_3_-SA_5_ and MMT_1_-SA_2_ microcapsule. Figure S3. TG-DSC curves for (A) MMT, (B)MMT_1_-SA_19_, (C) MMT_1_-SA_7_ (D) MMT_1_-SA_3_ and (E) MMT_3_-SA_5_. Figure S4. Zeta potentials of MMT1-SA4, MMT1-SA6, MMT1-SA8 in different pH. Figure S5. Cytotoxicity evaluation of MMT-SA to (A) HIEC-6 cells and (B) NCM-460 cells. Figure S6. FTIR of WGX-50, MMT-SA and WGX-50/MMT-SA microcapsule. Figure S7. TG (A) and DSC (B) of MMT, MMT-SA, WGX-50 and WGX-50/MMT-SA microcapsule. Table S1. The mixed mass ratios and labels of MMT-SA microcapsule. Table S2. In vitro release data of WGX-50. Table S3. Fitting results of WGX-50 release kinetics parameters using different models at pH 1.2, 6.8 and 7.4.

## References

[1] Peng S., Xu W., Liu H. (2023). Drug controlled releasing system based on polypyrrole modified multi-responsive hydrogel constructed from methacrylic acid and N-isopropylacrylamide. Colloid Surf. A Physicochem. Eng. Asp..

[2] Zhao H., Ye H., Zhou J., Tang G., Hou Z., Bai H. (2020). Montmorillonite-enveloped zeolitic imidazolate framework as a nourishing oral nano-platform for gastrointestinal drug delivery. ACS Appl. Mater. Interfaces.

[3] Chu J., Traverso G. (2022). Foundations of gastrointestinal-based drug delivery and future developments. Nat. Rev. Gastroenterol. Hepatol..

[4] Zarenezhad E., Marzi M., Abdulabbas H.T., Jasim S.A., Kouhpayeh S.A., Barbaresi S., Ahmadi S., Ghasemian A. (2023). Bilosomes as nanocarriers for the drug and vaccine delivery against gastrointestinal infections: Opportunities and challenges. J. Funct. Biomater..

[5] Arévalo-Pérez R., Maderuelo C., Lanao J. (2020). Recent advances in colon drug delivery systems. J. Control. Release.

[6] Azehaf H., Benzine Y., Tagzirt M., Skiba M., Karrout Y. (2023). Microbiota-sensitive drug delivery systems based on natural polysaccharides for colon targeting. Drug Discov. Today.

[7] Zheng J., Fan R., Wu H., Yao H., Yan Y., Liu J., Ran L., Sun Z., Yi L., Dang L. (2019). Directed self-assembly of herbal small molecules into sustained release hydrogels for treating neural inflammation. Nat. Commun..

[8] Tang M., Wang Z., Zhou Y., Xu W., Li S., Wang L., Wei D., Qiao Z. (2013). A novel drug candidate for Alzheimer’s disease treatment: Gx-50 derived from zanthoxylum bungeanum. J. Alzheimers Dis..

[9] Jaberifard F., Arsalani N., Ghorbani M., Mostafavi H. (2022). Incorporating halloysite nanotube/carvedilol nanohybrids into gelatin microsphere as a novel oral pH-sensitive drug delivery system. Colloid Surf. A Physicochem. Eng. Asp..

[10] Sun J., Xu Z., Hou Y., Yao W., Fan X., Zheng H., Piao J., Li F., Wei Y. (2022). Hierarchically structured microcapsules for oral delivery of emodin and tanshinone IIA to treat renal fibrosis. Int. J. Pharm..

[11] Wang X., Gao S., Yun S., Zhang M., Peng L., Li Y., Zhou Y. (2022). Microencapsulating alginate-based polymers for probiotics delivery systems and their application. Pharmaceuticals.

[12] Yu C., Naeem A., Liu Y., Guan Y. (2023). Ellagic acid inclusion complex-loaded hydrogels as an efficient controlled release system: Design, fabrication and in vitro evaluation. J. Funct. Biomater..

[13] Kaushik A.C., Kumar A., Deng Z., Khan A., Junaid M., Ali A., Bharadwaj S., Wei D. (2019). Evaluation and validation of synergistic effects of amyloid-beta inhibitor–gold nanoparticles complex on Alzheimer’s disease using deep neural network approach. J. Mater. Res..

[14] Prakash J., Kumar T., Venkataprasanna K., Niranjan R., Kaushik M., Samal D., Venkatasubbu G. (2019). PVA/alginate/hydroxyapatite films for controlled release of amoxicillin for the treatment of periodontal defects. Appl. Surf. Sci..

[15] Yang I., Chen Y., Li J., Liang Y.J., Lin T., Jakfar S., Thacker M., Wu S., Lin F. (2021). The development of laminin-alginate microspheres encapsulated with Ginsenoside Rg1 and ADSCs for breast reconstruction after lumpectomy. Bioact. Mater..

[16] Wang J., Deng H., Sun Y., Yang C. (2020). Montmorillonite and alginate co-stabilized biocompatible pickering emulsions with multiple-stimulus tunable rheology. J. Colloid Interface Sci..

[17] Dattilo M., Patitucci F., Prete S., Parisi O.I., Puoci F. (2023). Polysaccharide-based hydrogels and their application as drug delivery systems in cancer treatment: A review. J. Funct. Biomater..

[18] Yadav H., Agrawal R., Panday A., Patel J., Maiti S. (2022). Polysaccharide-silicate composite hydrogels: Review on synthesis and drug delivery credentials. J. Drug Deliv. Sci. Technol..

[19] Yuan Y., Xu X., Gong J., Mu R., Li Y., Wu C., Pang J. (2019). Fabrication of chitosan-coated konjac glucomannan/sodium alginate/graphene oxide microspheres with enhanced colon-targeted delivery. Int. J. Biol. Macromol..

[20] Li W., Chen J., Zhao S., Huang T., Ying H., Trujillo C., Molinaro G., Zhou Z., Jiang T., Liu W. (2022). High drug-loaded microspheres enabled by controlled in-droplet precipitation promote functional recovery after spinal cord injury. Nat. Commun..

[21] Nielsen R.B., Kahnt A., Dillen L., Wuyts K., Snoeys J., Nielsen U.G., Holm R., Nielsen C.U. (2019). Montmorillonite-surfactant hybrid particles for modulating intestinal P-glycoprotein-mediated transport. Int. J. Pharm..

[22] Gaharwar A., Cross L., Peak C., Gold K., Carrow J., Brokesh A., Singh K. (2019). 2D nanoclay for biomedical applications: Regenerative medicine, therapeutic delivery, and additive manufacturing. Adv. Mater..

[23] Khatoona N., Chu M., Zhou C. (2020). Nanoclay-based drug delivery systems and their therapeutic potentials. J. Mat. Chem..

[24] Ayazi H., Akhavan O., Raoufi M., Varshochian R., Motlagh N., Atyabi F. (2020). Graphene aerogel nanoparticles for in-situ loading/pH sensitive releasing anticancer drugs. Colloid Surf. B Biointerfaces.

[25] Wang T., Yi W., Zhang Y., Wu H., Fan H., Zhao J., Wang S. (2023). Sodium alginate hydrogel containing platelet-rich plasma for wound healing. Colloid Surf. B Biointerfaces.

[26] Dong X., Li Y., Huang G., Xiao J., Guo L., Liu L. (2021). Preparation and characterization of soybean Protein isolate/chitosan/sodium alginate ternary complex coacervate phase. LWT Food Sci. Technol..

[27] Wang W., Ni J., Chen L., Ai Z., Zhao Y., Song S. (2020). Synthesis of carboxymethyl cellulose-chitosan-montmorillonite nanosheets composite hydrogel for dye effluent remediation. Int. J. Biol. Macromol..

[28] You Y., Qu K., Huang Z., Ma R., Shi C., Li X., Liu D., Dong M., Guo Z. (2019). Sodium alginate templated hydroxyapatite/calcium silicate composite adsorbents for efficient dye removal from polluted water. Int. J. Biol. Macromol..

[29] Da Silva Fernandes R., de Moura M.R., Glenn G.M., Aouada F.A. (2018). Thermal, microstructural, and spectroscopic analysis of Ca^2+^ alginate/clay nanocomposite hydrogel beads. J. Mol. Liq..

[30] Ahamed A.F., Manimohan M., Kalaivasan N. (2022). Fabrication of Biologically Active Fish Bone Derived Hydroxyapatite and Montmorillonite Blended Sodium Alginate Composite for In-Vitro Drug Delivery Studies. J. Inorg. Organomet. Polym. Mater..

[31] Zhong L., Hu S., Yang X., Yang M., Zhang T., Chen L., Zhao Y., Song S. (2021). Difference in the preparation of two-dimensional nanosheets of montmorillonite from different regions: Role of the layer charge density. Colloid Surf. A Physicochem. Eng. Asp..

[32] Guo H., Qin Q., Chang J.-S., Lee D.-J. (2023). Modified alginate materials for wastewater treatment: Application prospects. Bioresour. Technol..

[33] Xu P., Song J., Dai Z., Xu Y., Li D., Wu C. (2021). Effect of Ca^2+^ cross-linking on the properties and structure of lutein-loaded sodium alginate hydrogels. Int. J. Biol. Macromol..

[34] Sharifzadeh G., Hezaveh H., Muhamad I., Hashim S., Khairuddin N. (2020). Montmorillonite-based polyacrylamide hydrogel rings for controlled vaginal drug delivery. Biomater. Adv..

[35] Zhao S., Li Y., Liu Q., Li S., Cheng Y., Cheng C., Sun Z., Du Y., Butch C., Wei H. (2020). An orally administered CeO_2_@Montmorillonite nanozyme targets inflammation for inflammatory bowel disease therapy. Adv. Funct. Mater..

[36] García-Guzmán P., Medina-Torres L., Calderas F., Bernad-Bernad M.J., Gracia-Mora J., Marcos X., Correa-Basurto J., Núñez-Ramírez D.M., Manero O. (2019). Rheological mucoadhesion and cytotoxicity of montmorillonite clay mineral/hybrid microparticles biocomposite. Appl. Clay Sci..

[37] (2009). Biological Evaluation of Medical Devices—Part 5: Tests for In Vitro Cytotoxicity.

[38] Christoforidou T., Giasafaki D., Andriotis E.G., Bouropoulos N., Theodoroula N.F., Vizirianakis I.S., Steriotis T., Charalambopoulou G., Fatouros D.G. (2021). Oral Drug Delivery Systems Based on Ordered Mesoporous Silica Nanoparticles for Modulating the Release of Aprepitant. Int. J. Mol. Sci..

[39] Li X., Zhang C., Wu S., Chen X., Mai J., Chang M.W. (2019). Precision Printing of Customized Cylindrical Capsules with Multifunctional Layers for Oral Drug Delivery. ACS Appl. Mater. Interfaces.

[40] Jing H., Huang X., Du X., Mo L., Ma C., Wang H. (2022). Facile synthesis of pH-responsive sodium alginate/carboxymethyl chitosan hydrogel beads promoted by hydrogen bond. Carbohydr. Polym..

