# Supplementary material for: Montmorillonite-Sodium Alginate Oral Colon-Targeting Microcapsule Design for WGX-50 Encapsulation and Controlled Release in Gastro-Intestinal Tract
Source: J Funct Biomater. 2023 Dec 19;15(1):3. doi: 10.3390/jfb15010003 (PMC10816513; doi:10.3390/jfb15010003)
Supplement: Supplementary file 1 [file jfb-15-00003-s001.zip › jfb-2701572-supplementary.pdf]

Journal of Functional Biomaterials

Supplementary Materials

Montmorillonite-Sodium Alginate Oral Colon-Targeting Microcapsule Design for WGX-50 Encapsulation and Controlled Release in Gastro-Intestinal Tract

Yibei Jiang [1], Zhou Wang [1], Ke Cao [2], Lu Xia [3], Dongqing Wei [4,*] and Yi Zhang [1,*]

[1] Department of Inorganic Materials, School of Minerals Processing and Bioengineering, Central South University, Changsha 410083, China; 215611016@csu.edu.cn (Y.J.); zhouwang@csu.edu.cn (Z.W.)
[2] Department of Oncology, The Third Xiangya Hospital of Central South University, Changsha 410078, China; csucaoke@163.com
[3] Center for Medical Genetics & Hunan Key Laboratory of Medical Genetics, School of Life Sciences, Central South University, Changsha 410078, China; xialu@sklmg.edu.cn
[4] State Key Laboratory of Microbial Meta bolism, School of Life Sciences and Biotechnology, Shanghai Jiao Tong University, Shanghai 200240, China
* Correspondence: yee_z10@csu.edu.cn (Y.Z.); dqwei@sjtu.edu.cn (D.W.)

Citation: Jiang, Y.; Wang, Z.; Cao, K.; Xia, L.; Wei, D.; Zhang, Y. Montmorillonite-Sodium Alginate Oral Colon-Targeting Microcapsule Design for WGX-50 Encapsulation and Controlled Release in Gastro-Intestinal Tract. *J. Funct. Biomater.* **2023**, *15*, 3. https://doi.org/10.3390/jfb15010003

Academic Editor: Pankaj Vadgama

Received: 20 October 2023
Revised: 9 December 2023
Accepted: 11 December 2023
Published: 19 December 2023

Publisher's Note: MDPI stays neutral with regard to jurisdictional claims in published maps and institutional affiliations.

Copyright: © 2023 by the authors. Submitted for possible open access publication under the terms and conditions of the Creative Commons Attribution (CC BY) license (https://creativecommons.org/licenses/by/4.0/).

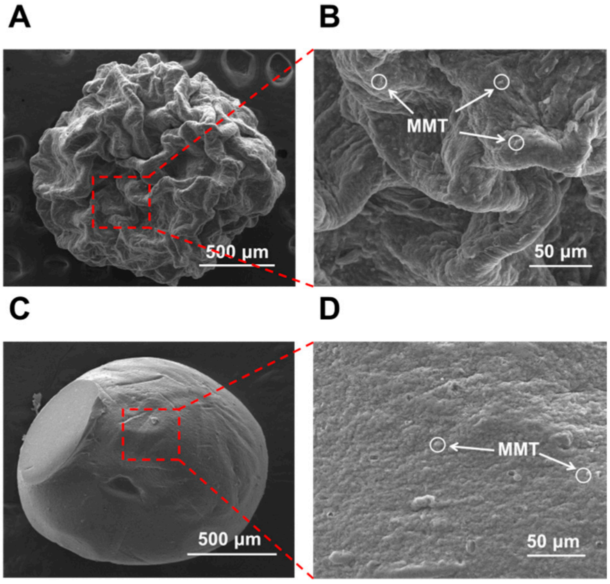

Figure S1. Freeze-dried microcapsules (**A**–**B**) and oven-dried microcapsules (**C**–**D**).

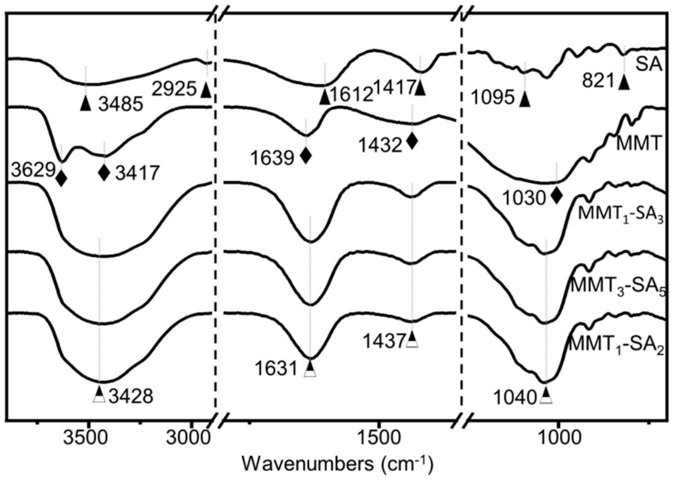

Figure S2. FTIR of SA, MMT, MMT_1-SA_3, MMT_3-SA_5 and MMT_1-SA_2 microcapsule.

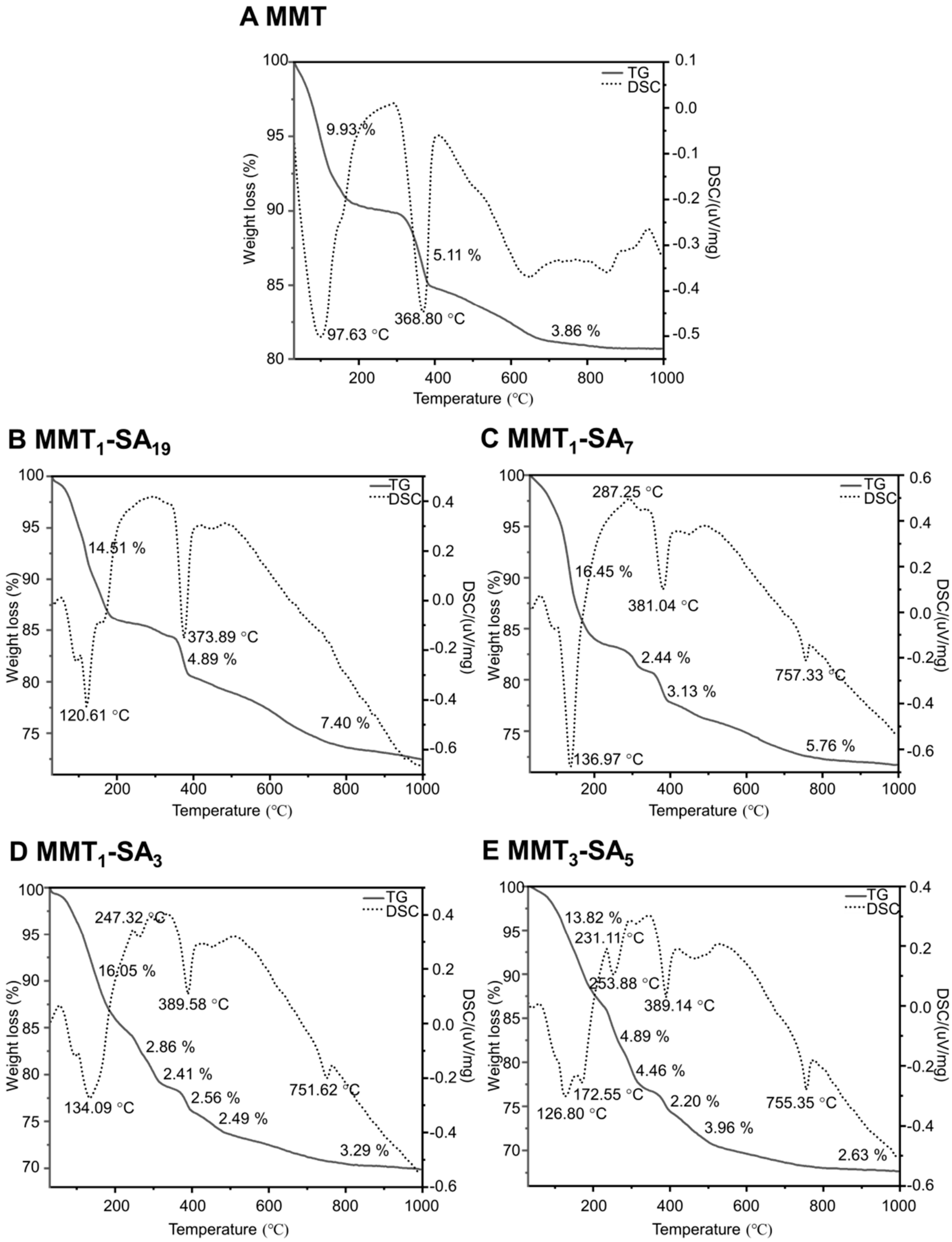

Figure S3. TG-DSC curves for (**A**) MMT, (**B**)MMT_1-SA_{19}, (**C**) MMT_1-SA_7 (**D**) MMT_1-SA_3 and (**E**) MMT_3-SA_5.

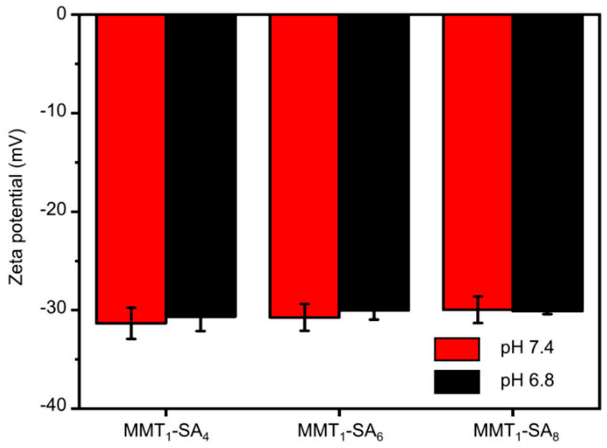

Figure S4. Zeta potentials of MMT_1-SA_4, MMT_1-SA_6, MMT_1-SA_8 in different pH.

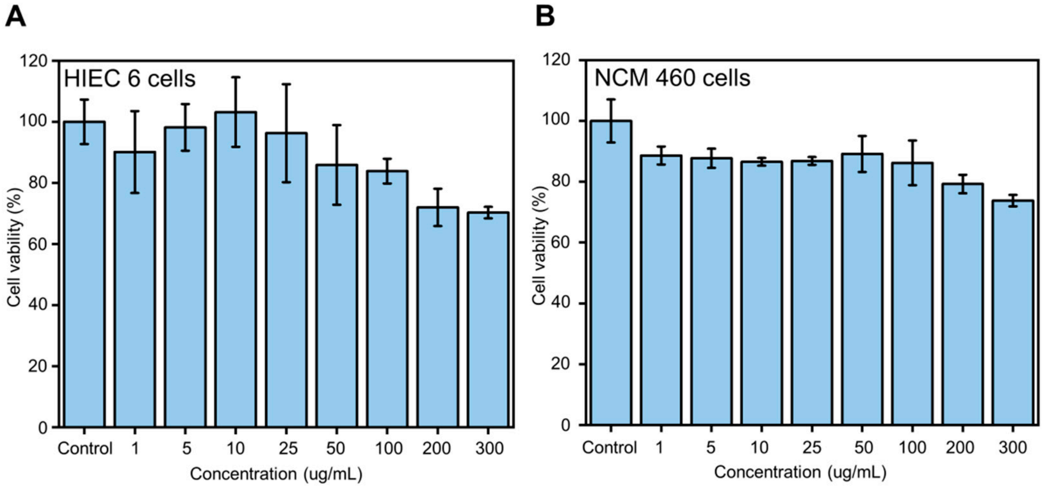

Figure S5. Cytotoxicity evaluation of MMT-SA to (**A**) HIEC-6 cells and (**B**) NCM-460 cells.

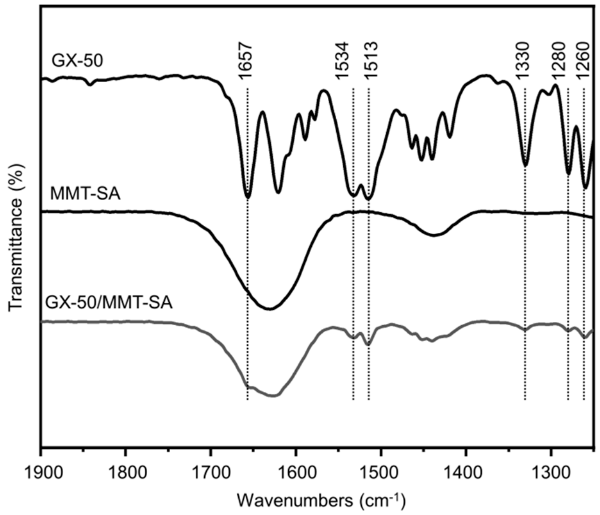

Figure S6. FTIR of WGX-50, MMT-SA and WGX-50/MMT-SA microcapsule.

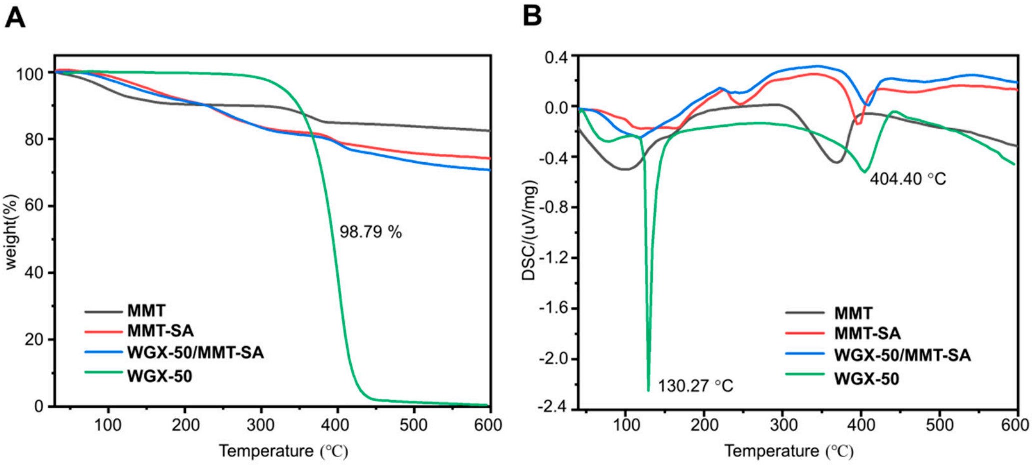

Figure S7. TG (**A**) and DSC (**B**) of MMT, MMT-SA, WGX-50 and WGX-50/MMT-SA microcapsule.

Table S1. The mixed mass ratios and labels of MMT-SA microcapsule.

Samples	Sodium Alginate(mg)	MMT(mg)
MTT	0	200
MMT_1-SA_{19}	10	190
MMT_1-SA_7	25	175
MMT_1-SA_3	50	150
MMT_3-SA_5	75	125
MMT_1-SA_2	75	150
MMT_1-SA_4	75	300
MMT_1-SA_6	75	450
MMT_1-SA_8	75	600

Table S2. In vitro release data of WGX-50.

Theoretical value of WGX-50 (mg)	WGX-50 Encapsulation (mg)	WGX-50/MMT-SA (mg)	EE (%)	SD	DL (%)	SD
25	24.81	435.8	99.25	1.25E-4	5.71	5.57E-4
50	49.80	493.5	99.60	1.38E-4	10.09	1.40E-3
100	99.62	497.2	99.62	9.65E-5	20.04	2.89E-3
200	199.34	634.43	99.67	2.31E-5	31.43	6.53E-3

Table S3. Fitting results of WGX-50 release kinetics parameters using different models at pH 1.2, 6.8 and 7.4.

Release condition	Zero-order		First-order		Higuchi		Korsmeyer-Peppas	
	R^2	k	R^2	k	R^2	k	R^2	n
pH 1.2	0.985	0.0321	0.978	5.540E-5	0.990	0.501	0.980	1.062
pH 6.8	0.917	0.0171	0.774	3.046E-6	0.941	5.122	0.881	1.580
pH 7.4	0.0255	4.210E-4	0.719	0.00857	0.0628	0.0404	0.122	0.013
